# Elucidating the 3D Structure of a Surface Membrane Antigen from *Trypanosoma cruzi* as a Serodiagnostic Biomarker of Chagas Disease

**DOI:** 10.3390/vaccines10010071

**Published:** 2022-01-03

**Authors:** Flavio Di Pisa, Stefano De Benedetti, Enrico Mario Alessandro Fassi, Mauro Bombaci, Renata Grifantini, Angelo Musicò, Roberto Frigerio, Angela Pontillo, Cinzia Rigo, Sandra Abelli, Romualdo Grande, Nadia Zanchetta, Davide Mileto, Alessandro Mancon, Alberto Rizzo, Alessandro Gori, Marina Cretich, Giorgio Colombo, Martino Bolognesi, Louise Jane Gourlay

**Affiliations:** 1 Department of Biosciences, Università degli Studi di Milano, Via Celoria 26, 20133 Milano, Italy; DiPisa.Flavio@hsr.it (F.D.P.); stefano.debenedetti@unimi.it (S.D.B.); martino.bolognesi@unimi.it (M.B.); 2 Consiglio Nazionale delle Ricerche, Istituto di Scienze e Tecnologie Chimiche “Giulio Natta” (SCITEC), Via Mario Bianco 9, 20131 Milano, Italy; enrico.fassi@unimi.it (E.M.A.F.); angelo.musico94@gmail.com (A.M.); roberto.frigerio94@gmail.com (R.F.); alessandro.gori@cnr.it (A.G.); marina.cretich@cnr.it (M.C.); g.colombo@unipv.it (G.C.); 3 Dipartimento di Scienze Farmaceutiche, Università degli Studi di Milano, Via L. Mangiagalli 25, 20133 Milano, Italy; 4 Istituto Nazionale Genetica Molecolare, Padiglione Romeo ed Enrica Invernizzi, IRCCS Ospedale Maggiore Policlinico, 20122 Milano, Italy; bombaci@ingm.org (M.B.); grifantini@ingm.org (R.G.); 5 PRIMM S.r.l., Via Fatebenefratelli 15, 20121 Milano, Italy; angela.pontillo@primm.it; 6 PRIMM S.r.l., Via dell’Artigianato 2, Dosson di Casier, 31030 Treviso, Italy; rigocinzia4@gmail.com (C.R.); sa.esse.sa@gmail.com (S.A.); 7 UOC Microbiologia Clinica, Virologia e Diagnostica delle Bioemergenze, ASST FBF Sacco, Via G.B Grassi, 74, 20157 Milano, Italy; grande.romualdo@asst-fbf-sacco.it (R.G.); nadia.zanchetta@asst-fbf-sacco.it (N.Z.); davide.mileto@unimi.it (D.M.); alessandro.mancon@unimi.it (A.M.); alberto.rizzo@asst-fbf-sacco.it (A.R.); 8 Dipartimento di Chimica, Università di Pavia, V.le Taramelli 12, 27100 Pavia, Italy; 9 Centro di Ricerca Pediatrica Romeo ed Enrica Invernizzi, Università degli Studi di Milano, 20133 Milano, Italy

**Keywords:** Chagas disease, *Trypanosoma cruzi*, neglected tropical disease, structural vaccinology, peptide microarray, in silico epitope mapping, immunodiagnostics

## Abstract

Chagas disease (CD) is a vector-borne parasitosis, caused by the protozoan parasite *Trypanosoma cruzi*, that affects millions of people worldwide. Although endemic in South America, CD is emerging throughout the world due to climate change and increased immigratory flux of infected people to non-endemic regions. Containing of the diffusion of CD is challenged by the asymptomatic nature of the disease in early infection stages and by the lack of a rapid and effective diagnostic test. With the aim of designing new serodiagnostic molecules to be implemented in a microarray-based diagnostic set-up for early screening of CD, herein, we report the recombinant production of the extracellular domain of a surface membrane antigen from *T. cruzi* (*Tc*SMP) and confirm its ability to detect plasma antibodies from infected patients. Moreover, we describe its high-resolution (1.62 Å) crystal structure, to which in silico epitope predictions were applied in order to locate the most immunoreactive regions of *Tc*SMP in order to guide the design of epitopes that may be used as an alternative to the full-length antigen for CD diagnosis. Two putative, linear epitopes, belonging to the same immunogenic region, were synthesized as free peptides, and their immunological properties were tested in vitro. Although both peptides were shown to adopt a structural conformation that allowed their recognition by polyclonal antibodies raised against the recombinant protein, they were not serodiagnostic for *T. cruzi* infections. Nevertheless, they represent good starting points for further iterative structure-based (re)design cycles.

## 1. Introduction

American trypanosomiasis, or Chagas disease (CD), is a devastating disorder that affects millions of people worldwide, leading to around 50,000 deaths per year [1,2]. To date, it is estimated that around 8 million people in Central America, South America, and Mexico, especially in rural areas, are affected by CD, most being unaware of their condition [3]. Another 65–100 million people live in areas at risk of infection around the world. In recent decades, due to climate changes and increased migration, incidence of CD is increasing in Europe, North America, and in the western Pacific region, including Japan and Australia. In these situations, transmission of CD occurs mainly through vertical transmission from mother to child, blood transfusion, and organ transplantation [4].

In contrast, in endemic regions, the main causative agent of CD is the kinetoplastid protozoan *Trypanosoma cruzi* (TC) that infects humans via an intermediate vector, the triatomine bug of the Reduviidae family, commonly called the kissing bug [5]. In the insect gut, the parasite replicates in an epimastigote form, before evolving into a metacyclic trypomastigote. When the kissing bug ingests a blood meal, the infective stage starts. The *T. cruzi* parasite is excreted in the feces that are deposited by the bug and penetrates the human host via skin lesions or mucosal surfaces. Once in the blood, the metacyclic trypomastigote infects the host cells, firstly evolving into an aflagellate amastigote in the cytoplasm, before replication via binary fission, and final transformation into a flagellated trypomastigote, which is released from the cell to circulate and infect other cells. The cycle is complete when a triatomine takes up circulating parasites during a blood meal [3,5].

The infection proceeds via two stages: the first acute phase is generally asymptomatic or characterized by mild manifestations such as swelling around the site of inoculation, fever, or tachycardia that disappear spontaneously. This phase is characterized by a high-grade parasitemia [6]. After a lapse of time up to 8 weeks, circulating parasite levels rapidly decrease and the chronic stage starts. The majority of infected people (60–70% of cases) enter a prolonged asymptomatic stage, which can remain clinically silent for their whole life (defined as chronic asymptomatic or indeterminate) [7,8]. An estimated 30–40% of the infected patients can develop severe and sometimes life-threatening clinical manifestations such as cardiac, cardio-digestive, or neurological disorders [9].

Current therapies for CD are limited to symptomatic and parasite-specific treatments [4,10]. Two currently used FDA-approved drugs, benznidazole and nifurtimox, involve long treatment durations and are hindered by several side effects, including drug toxicity, ineffective drug delivery, or cost [11], underlining the necessity to develop improved and affordable drugs [12,13,14,15].

In terms of diagnosis, the human CD can be diagnosed at any stage of the disease [16]. In the acute phase, the presence of the circulating parasite can be detected by parasitological tests, including identification of trypomastigotes through blood smear by microscopy, by multiplication as blood cultures, or by PCR [17]. These methods are not applicable during the chronic phase, when the levels of circulating parasites are consistently lower. In this later stage, the disease can be diagnosed by detecting the α-*T. cruzi* IgG antibodies using conventional serology methods (i.e., hemagglutination assay, ELISA, or indirect immunofluorescence). In addition, hepatogram, electrocardiogram (ECG), chest radiography, or routine lab tests can be performed for both acute and chronic phases for a more accurate clinical evaluation [18,19].

Despite the availability of different types of detection methods for CD diagnosis, there are several reasons to develop new immunodiagnostic tools for early and effective diagnosis of the disease. Namely, current therapy cannot prevent tissue damage progression in later symptomatic (cardiac and or digestive) stages of the disease. Therefore, early diagnosis is essential to initiate treatment and improve patient prognosis. Moreover, as recently demonstrated by COVID-19, generation of new, rapid, and highly sensitive point-of-care diagnostic tests is paramount to understand epidemiology and to control the spread of infectious diseases in a high-throughput context, especially with the increasing ease of travel and migration.

Therefore, the production of recombinant *Trypanosoma* spp. protein antigens and/or synthetic peptide-based epitope microarrays have potential applications for the serological diagnosis of CD. In this context, reverse vaccinology (RV) and structural vaccinology (SV) play an important role in facilitating the identification and engineering of new serodiagnostic antigens, respectively [20,21]. Although born in the field of vaccine design, these methodologies, flanked by computational biology epitope design methods, have been demonstrated to be pivotal for serodiagnostic applications [20,22,23]. Starting from the 3D structures of known serodiagnostic antigens, in silico epitope mapping approaches can help pinpoint the most immunoreactive portions of an antigen that, when synthetized as synthetic peptides, can be used to capture antigen-specific antibodies from serum samples [24]. Epitope-based peptide microarrays represent appealing tools compared with full-length antigen-based chips, allow the fast screening of thousands of different epitope peptides with higher sensitivity and specificity, without the drawbacks associated with full-length proteins (e.g., high costs, stability issues, storage constraints, cross-reactivity). In addition, epitope peptide microarrays can be fine-tuned with ad hoc epitopes that could detail the status of infection [25] and discriminate between different pathogens and variants, or with epitope probes derived from multiple pathogens. In this last case, a single diagnostic panel could be used to identify different pathologies in one shot.

In this context, we describe the production and high-resolution (1.62 Å) crystal structure determination of a surface membrane protein from *T. cruzi* (*Tc*SMP) as a potential serodiagnostic antigen for structure-based in silico epitope mapping and design. *Tc*SMP is a poorly characterized protein, reported to be involved in mammalian cell invasion, with an auxiliary role to gp82, the major adhesion protein of metacyclic trypomastigotes [26]. It is highly conserved among different *Trypanosoma* spp., making it an ideal candidate for CD diagnosis. In fact, in this pilot study, we demonstrate the ability of recombinant *Tc*SMP to detect serum IgGs from CD infected subjects, residing in the Lombardy area of Italy, confirming its potential as a diagnostic biomarker. In addition, on the basis of the crystal structure, structure-guided epitope predictions revealed a particularly immunogenic region of the protein housing residues that were designed as two linear epitope peptides. Although shown to adopt the correct structural conformation reflected in the recombinant protein antigen, they did not detect plasma antibodies in infected patients, suggesting that further cycles of epitope redesign should be undertaken.

## 2. Materials and Methods

### 2.1. Protein Expression and Purification

To facilitate the soluble, heterologous production of *Tc*SMP, we cloned only residues 39 to 263, corresponding to the extracellular domain, into pET14 for recombinant expression in bacterial cells. The codon optimized gene sequence, encoding for the extracellular domain (residues 39 to 263) of *Trypanosoma cruzi* surface membrane protein TcSMP11.90 (Uniprot code Q4DZR9), was chemically synthesized and cloned into the pET14b vector (Biomatik, Cambridge, Ontario, Canada) via *NdeI* and *BamHI* restriction sites, incorporating a N-terminal histidine tag and thrombin cleavage site onto the expressed protein. Due to the presence of six cysteine residues, *Tc*SMP was expressed in SHuffle^®^ Express bacterial cells (New England Biolabs, MA, USA) in Luria Bertani broth supplemented with 100 μg/mL of ampicillin. Cells were grown at 30 °C with shaking at 210 rpm until an OD_600nm_ of 0.6 was reached. Protein overexpression was induced by addition of 0.5 mM isopropyl β-D-1-thioglagropyranoside (IPTG), with further incubation for 16 h at 16 °C, with shaking at 220 rpm. Cells were harvested at 6500 rpm at 4 °C, then resuspended in binding buffer (10 mM Tris-HCl pH 8.0, 50 mM NaCl, 20 mM imidazole) and lysed at a constant pressure (25,000 lb/in^2^) with a cell disruptor (Constant Systems Ltd., Daventry, UK).

The supernatant was collected by centrifugation at 18,000 rpm for 30 min and loaded onto a 1 mL HisTrap HP column (Cytiva, Marlborough, Massachusetts, United States), then pre-equilibrated in binding buffer using an ÄKTA Explorer System (Cytiva). The purified protein was eluted in elution buffer (10 mM Tris-HCl pH 8.0, 50 mM NaCl, 250 mM imidazole), following an intermediate wash step in the same buffer, containing 50 mM imidazole to remove non-specifically bound proteins. Fractions containing purified protein were analyzed by SDS-PAGE on precast 12% ExpressPlus polyacrylamide gel (GenScript, Piscataway, New Jersey, United States), pooled, and dialyzed overnight against crystallization buffer (10 mM Tris-HCl pH 8.0, 50 mM NaCl) at 4 °C. Histidine tag cleavage was induced in the overnight dialysis by adding 5 U thrombin (Cytiva) per milligram of *Tc*SMP protein.

Size exclusion chromatography was performed on a Superdex 75 (10/300) GL column (Cytiva) pre-equilibrated with crystallization buffer. *Tc*SMP was eluted by isocratic elution at a flow rate of 0.3 mL/min. Purified *Tc*SMP was concentrated to 5 mg/mL for crystallization trials using an Amicon Ultra Centrifugal filter unit with a MW cut-off of 3 kDa (Millipore, Burlington, MA, USA). All chromatographic steps were performed at room temperature.

### 2.2. Crystallization

Purified *Tc*SMP was crystallized using an Oryx4 crystallization robot (Douglas Instruments, Hungerford, UK) using the sitting drop technique. Crystallization screens were performed at 20 °C, mixing the protein sample with crystallization reservoir solution, at three different protein concentrations (30%, 50%, 70%) in a final drop volume of 0.60 µL, equilibrated over 100 µL of reservoir solution containing each of the 96 prepared PACT Premier screen conditions (Molecular Dimensions Ltd., Newmarket, United Kingdom) in 96-well flat-bottomed CrystalQuick^TM^ plates (Greiner Bio-One Italia S.r.l., Cassina de’ Pecchi, Milano, Italy). Single and sizable crystals suitable for X-ray diffraction experiments grew overnight in condition 1–2 containing 0.1 M SPG buffer pH 5, 25% *(w*/*v)* PEG1500 (SPG: succinic acid, sodium dihydrogen phosphate and glycine at the molar ratio 2:7:7), and were directly cryo-cooled in liquid nitrogen for data collection.

### 2.3. X-ray Data Collection and Structure Determination

X-ray diffraction data were collected on a single *Tc*SMP crystal at 100K at the Diamond Light Source (DLS, Didcot, United Kingdom) on beamline I04, equipped with a Dectris Eiger2 XE 16M detector. *Tc*SMP reflections were reduced to unique reflections using XDS [27] and scaled with Scala [28] from the CCP4 suite [29].

The structure was solved by molecular replacement using MOLREP [30] and the crystal structure of *Trypanosoma brucei* Procyclic Specific Surface Antigen-2 (PDB ID 5KLH; [31]) as a search model (40% sequence identity with *Tc*SMP). The initial molecular replacement solution was subjected to subsequent cycles of manual building in Coot [32] and refinement with phenix.refine [33]. Water molecules were added with the program ARP/wARP [34] and visually inspected with Coot [32].

The final model was inspected and validated with the software Molprobity [35]. Structure solution and refinement statistics are reported in Table S1. Structure factors and atomic coordinates have been deposited in the PDB (www.rcsb.org, accessed on 7 February 2020) under entry code 6Y0D.

### 2.4. Human Sera Sample Collection

Human specimens were retrospectively selected from the Sacco Hospital biological repository, including those who tested positive for anti-*T. cruzi* antibodies (Abs; as negative controls, sera from Healthy Donors (HDs) were used). Blood was collected in silica-coated tubes with polymeric separation gel and centrifuged after clotting at 3000 rpm for 20 min. All samples were tested with ARCHITECT Chagas chemiluminescent assay on ARCHITECT i2000SR system (Abbott, Chicago, IL, USA) to determine the presence of anti-*T. cruzi* Abs.

### 2.5. Polyclonal Antibody Production

Polyclonal antibodies were raised in two New Zealand white rabbits and four mice cd1 immunized with 1.5 mg/mL *Tc*SMP (His-tag removed and prepared in 1X PBS). Briefly, preimmune sera were collected (1 mL/Rabbit–0.1 mL/Mouse) prior to the following immunization protocol: first immunization (200 μg Ag/rabbit and 20 μg/mouse in CFA—complete Freund adjuvant); after 14 days, a second immunization (100 μg Ag/rabbit and 10 μg/mouse in IFA—incomplete Freund adjuvant) and then two weekly immunizations with 100 μg Ag/rabbit and 10 μg/mouse in IFA. On the 28th day, a 500 μL/rabbit and 50 μL/mouse bleed was removed for ELISA test. On day 31, a final bleed was carried out (50 mL/rabbit–0.5 mL/mouse)—final bleed and a final ELISA test (T-end).

### 2.6. Dissociation-Enhanced Lanthanide Fluoroscence ImmunoAssays (DELFIA)

The DELFIA^®^ immunoassay is a time-resolved fluorescence method that can be used to study antibody binding to solid-phase proteins or peptides. A set of sera, comprising 9 *T. cruzi-*infected patient samples and 15 HD samples, was analyzed against recombinant *Tc*SMP. Purified recombinant *Tc*SMP (20 μg/mL) in 1X PBS was used to coat DELFIA plates (PerkinElmer). The serum assay procedure was automatically performed by the Freedom-EVO Liquid Hander (Tecan, Männedorf, Switzerland), as previously described [36]. In brief, plates were blocked for 1 h at 37 °C with a blocking reagent (Perkin Elmer Italia S.r.l., Milano, Italy). Serum samples were diluted 1:300 in 0.1% *(v*/*v)* TPBS containing 1% *(w*/*v)* BSA and incubated for 1 h at 37 °C. Plates were then washed four times with washing buffer (PerkinElmer) and incubated for 30 min at RT in the dark with Europium-labeled α-Human IgG serum (1:500 in diluting buffer, PerkinElmer). After extensive washing by Hydrospeed™ (Tecan), plates were left at RT for 10 min and then read on an Infinite F200 PRO instrument (Tecan). Fluorescence intensity values, higher than the mean of HD plus one standard deviation, were considered as positive. DELFIA results were analyzed using Student’s *t*-test for the MFI, as well as the two-sided Fisher’s exact test to concern the recognition frequency, using the GraphPad software 9.2.0.

### 2.7. Protein Microarrays

The immunoreactivity of *Tc*SMP was assessed on a protein microarray against a panel of immune sera from 8 patients positive for anti-*T. cruzi* Abs versus a panel of 14 healthy donor (HD) controls. Silicon slides were coated by MCP2 (Lucidant Polymers, Sunnyvale, CA, USA), as previously described [37,38]. *Tc*SMP was dissolved in PBS (1 mg/mL) and printed using a non-contact Spotter S12 (Scienion Co., Berlin, Germany). Printed slides were placed in a humid chamber overnight at room temperature. Slides were then blocked with Blocking solution (50 mM ethanolamine dissolved in water, pH 9) for 1 h, washed with distilled water, and dried under a nitrogen stream.

All serum samples were then diluted 1:10 in incubation buffer (50 mM Tris-HCl pH 7.6, 150 mM NaCl, 0.02% *(v*/*v)* TWEEN 20) with 1% *(w*/*v)* BSA and incubated dynamically for 1 h at room temperature. Slides were washed 3 times for 1 min with washing buffer (50 mM Tris-HCl pH 9, 250 mM NaCl, 0.05% *(v*/*v)* TWEEN 20). The second incubation with secondary antibody was performed with Anti-Human IgG-Cy3 (Jackson ImmunoResearch, West Grove, PA, USA) diluted 1:1000 in incubation buffer 1% *(w*/*v)* BSA. Finally, slides were washed and dried, and the analysis was performed by TECAN power scanner at 50% laser intensity and 100% gain. In total, 16 samples were tested (8 healthy serum samples and 8 samples affected by Trypanosomiasis).

### 2.8. Peptide Microarrays

Silicon slides were treated as previously described and printed with a 1 mg/mL Streptavidin solution dissolved in PBS. After blocking, slides were incubated with 500 µM PBS solution of biotinylated peptides in order to immobilize the peptides in a controlled orientation to the surface by the streptavidin-biotin binding. Slides were washed 3 times for 1 min with washing buffer. Immune and pre-immune sera from mouse and rabbit were diluted 1:50 in incubation buffer with 1% *(w*/*v)* BSA and incubated dynamically for 1 h at room temperature. Slides were washed 3 times for 1 min with washing buffer. The second incubation with secondary antibody was performed with anti-Mouse IgG-Cy3 and anti-Rabbit IgG-Cy3 (Jackson ImmunoReserarch, West Grove, PA, USA) diluted 1:1000 in incubation buffer 1% *(w*/*v)* BSA. Finally, slides were washed and dried, and the analysis was performed by TECAN power scanner at 50% laser intensity and 100% gain.

### 2.9. Molecular Dynamics Simulations

Three independent 500 ns long MD simulations (total 1.5 µs) were carried out on the *Tc*SMP crystal structure using the Amber16 software package, applying the Amber-ff14SB force field [39]. The TLEAP module of AmberTools16 was used to fully solvate the system in a TIP3P [40] water box and to add a proper number of counter ions in order to ensure the overall charge neutralization of the system. After minimization, the system was subjected to a multi-step equilibration phase where temperature was slowly increased to 300 K in 300 ps, and pressure was increased to 1 atm (see the Supporting Information for details). Finally, three independent replicas of 500 ns each (1.5 µs in total) of unrestrained simulations were run in an NPT ensemble, where temperature and pressure were kept constant, applying the Langevin thermostat [41] and Monte Carlo barostat [42], respectively. MD simulations were run with a time step of 2 fs using the PMEMD code in the GPU accelerated version [43]. Electrostatic interactions were evaluated by using the Particle Mesh Ewald method [44], setting a cut-off of 9 Å. During the calculations, all the bonds involving hydrogen atoms were constrained applying the SHAKE algorithm [45]. MD analyses were carried out on a meta-trajectory, obtained by concatenating all three independent trajectories. To define the structure on which to apply the MLCE epitope prediction, we applied the Daura et al. clustering procedure [46]. The root mean square fluctuation (RMSF), considering the crystal structure as reference, was calculated using the GROMACS (version 5.0.7) software package [47], including only backbone atoms.

### 2.10. Prediction of Epitopes: MLCE

Epitope predictions were performed through the matrix of local coupling energies (MLCE) method [48], which is able to combine the energetic profile of a given protein with assessment of its structural/dynamical determinants. The MLCE method can detect non-optimized/low intensity energetic interaction networks, which represent the protein portions more predisposed to interact with antibodies. This method has been extensively validated and described in the literature ([49,50,51], see the Supporting Information for details). In short, the algorithm selects the contiguous regions on the protein surface, which are considered to have minimal coupling energies with the rest of the structure (i.e., epitopes), on the basis of the eigenvalue decomposition of the matrix showing the non-bonded interaction of all residue pairs. Epitopes are identified by filtering of the simplified matrix with the contact matrix, and the selection is performed according to a threshold value (softness). It defines the percentage of the set of putative interaction sites by including the increasing residue–residue coupling values until the number of couplings (i.e., the lowest contact-filtered pairs under the threshold) were reached. In this article, MLCE analysis was carried out using the default prediction softness level (10%).

## 3. Results and Discussion

### 3.1. Expression and Purification of the Extracellular Domain of TcSMP

*Tc*SMP is a 401-residue surface protein with two transmembrane regions (residues 13 to 35 and residues 264 to 286), as predicted using the TMHMM server (http://www.cbs.dtu.dk/services/TMHMM/, accessed on 5 July 2019) [52]. To date, the function of TcSMP remains to be elucidated; however, knock-out studies carried out on a homologous antigen (43.7% sequence identity) from T. *brucei* (procyclic-specific surface antigen-2; *Tb*PSSA) underlined the importance of the protein for parasite transmission [53]. For diagnostic purposes, we focused on the extracellular domain of *Tc*SMP, which was expressed and purified to high purity (>95%), as described in the Section 2, with a final protein yield of 5 mg/L bacterial culture (Figure S1).

### 3.2. 3D Structure of TcSMP

The crystal structure of *Tc*SMP was solved at 1.62 Å resolution. Crystals belonged to the monoclinic C2 space group, with one molecule in the asymmetric unit and a Matthews coefficient of 2.01 Å^3^/Da (estimated solvent content of 39.5%). Overall, the electron density map was of good quality, covering residues from 41 to 254 with no gaps. Electron density for the first two and the last nine residues of the protein, as well as the N-terminal His tag, were absent due to flexibility of these regions.

**Figure 1:**
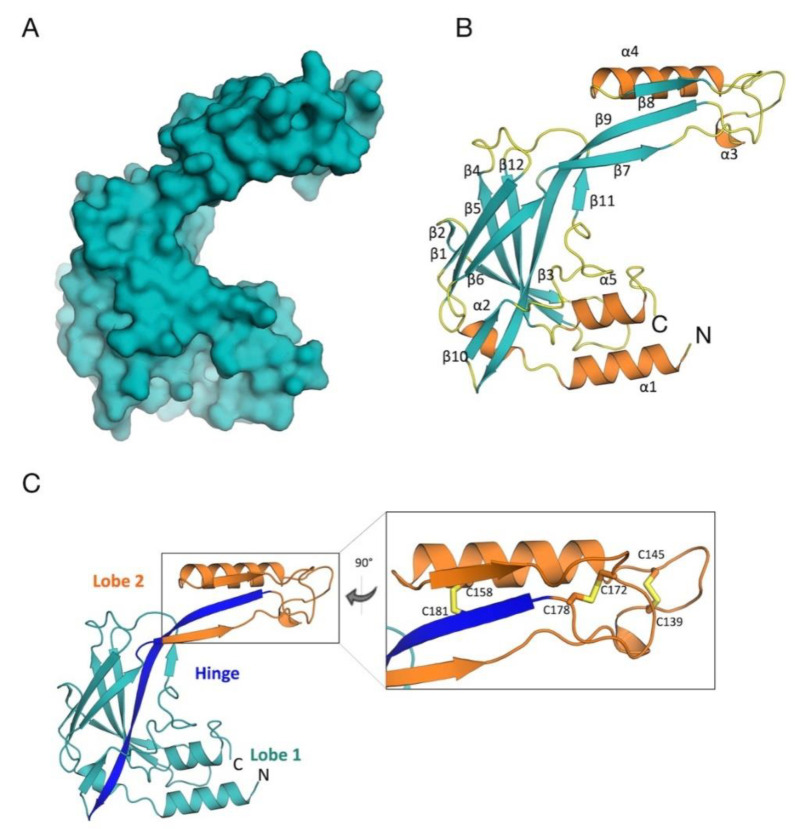
3D structure of *Tc*SMP. (**A**) Surface representation, illustrating the “C-shaped” organization of *Tc*SMP. (**B**) Cartoon, secondary structure representation of *Tc*SMP (β-strands in teal, α-helices in orange, 3-, 4-, and 5-turns and loops in yellow). The N and C-termini are indicated. (**C**) Stereo view of the *Tc*SMP crystal structure. *Tc*SMP is structurally organized into two lobes (colored in teal and orange), connected by a hinge portion, constituted by a β-strand and a connecting loop, depicted in blue. A detailed view of the region of lobe 2 that hosts the three disulfide bonds (sticks) is shown. This figure was generated using CCP4mg [54].

The 3D crystal structure of *Tc*SMP displayed an elongated, “C-shaped” architecture, comprising two lobes (lobes 1 and 2), linked together by a flexible hinge region. Overall, the structure was found to be composed of 12 β-strands and 5 short α-helices with an elongated strand (β9) that stretches from lobe 2 across to lobe 1. The two lobes are also connected by a loop located between strands β6 and β7 (Figure 1).

Interestingly, a short peptide was observed to be bound to the hinge region in a structurally homologous antigen from *Trypanosoma congolese* (*Tc*ISA, pdb code 5KMX, [31]), suggesting a ligand binding role for this region, although the physiological target has yet to be revealed [53]. On the basis of the confirmed role of *Tb*PSSA-2 in parasite transmission and the ligand binding site observed in TcPSSA, Fragoso et al. proposed a sensory role for *Tb*PSSA-2 in parasite division, differentiation, and migration [53].

The larger lobe, lobe 1, comprised residues from both the amino and carboxy termini, and hosted most of the secondary structural elements of the protein. It presented a β-strand core (β1–6, β10 and β12) and peripheral helices (α1 and α5), plus helix α2, located between helix α1 and strand β1. This core region, rich in β-sheets, was found to confer great stability to the protein; in particular β9, β12, and β4 on one side, and β5 and β6 on the other, forming extensive hydrogen bond patterns, attributing strong inter-strand stability.

Lobe 2 comprised β-strands β7, β8, and β11 and the elongated connector β9 strand, plus helices α3 and α4. Strands β7, β8, and β9 formed an antiparallel β-sheet. This lobe was found to be stabilized by intra-molecular hydrogen bonds between residues coming from β7 and β9 strands, as well as by three disulfide bridges, formed by C139–C145, C158–C181, and C172–C178 (Figure 1). As described further, these disulfide bridges pertain to the two antigenic regions of the protein, detected by in silico epitope mapping.

With regards to homology with other proteins of known structure, 3D structure-based comparisons performed with the DALI server (http://ekhidna2.biocenter.helsinki.fi/dali/, accessed on 4 June 2021 [55] revealed that *Tc*SMP exhibits structural similarity with two other antigens from *Trypanosoma* species: the *T. brucei* procyclic-specific surface antigen-2 (*Tb*PSSA-2:pdb code 5KLH, [31]) and *T. congolense* insect stage antigen (*Tc*ISA; pdb code 5KMX, [31]) (see the Supplementary Information). As determined by the secondary structure matching (SSM) program in Coot, superposition with the *T. brucei* antigen (see Figure S2) revealed a high degree of structural similarity (RMSD of 1.9 Å over 162 aligned Cα atoms), with large differences only in the lobe 2 region, whereas structural alignment with the antigen from *T. congolense* resulted in an overall higher RMSD value (3.3 Å) with major deviations in lobe 2 and helices α1 and α5 of lobe 1.

The lack of a human homolog may highlight *Tc*SMP as a potential therapeutic target for the development of parasite-specific drugs that are effective also against other *Trypanosoma* species.

### 3.3. In Silico Epitope Predictions

3D structure information on antigens can be used to predict the location of epitopes that trigger B-cell and/or T-cell immune responses [56]. Such information can help aid the design of peptide-based mimics, epitope-containing protein domains, or completely new antigens that can have applications as diagnostic biomarkers and or vaccine components [20]. Furthermore, often, designed epitopes can possess improved immunological properties with respect to the cognate antigen [20].

**Figure 2:**
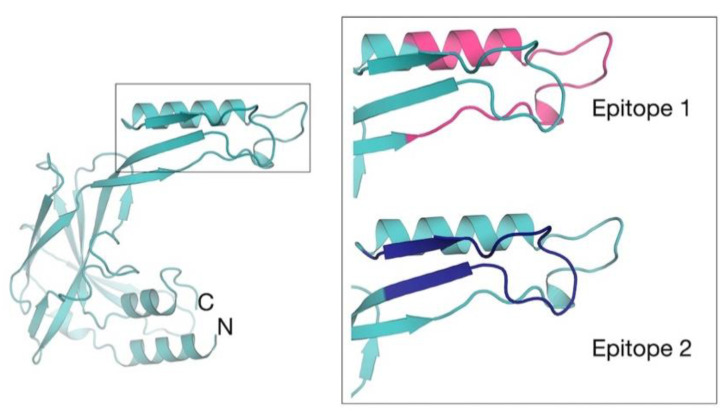
Structure-based *Tc*SMP epitope mapping. The location of computationally predicted epitopes are mapped on the crystal structure of *Tc*SMP. A zoom view of epitope 1 (residues 130–156; pink) and epitope 2 (residues 163–182; blue) is shown. This figure was generated using CCP4mg [54].

In this context, on the basis of the *Tc*SMP crystal structure, we performed in silico epitope predictions to identify the most immunoreactive portion(s) of the protein (i.e., more prone to be recognized by antibodies). Epitope identification aims to inspire the development of synthetic peptides with improved immunological properties. Two separate, sequential linear epitopes (Ep1: residues 130 to 156; Ep2: residues 163 to 182) were predicted and located to lobe 2 (Figure 2). The root mean square fluctuation (RMSF) was calculated, considering the entire MD simulations length, in order to ascertain whether predicted epitopes were actually located in dynamic regions of the protein (i.e., more prone to interact with an antibody). Our results show that both predicted epitopes corresponded to RMSF peaks (i.e., portions of the protein that fluctuate and can suitably be engaged by antibodies in immune responses). In particular, Ep1 was found to be located in correspondence of the most dynamic protein region (Figure S3). Given their close proximity to one another in both sequence and conformational space, they may constitute a single, large conformational epitope. Both epitopes are stabilized by disulfide bonds. Ep1 is stabilized by two disulfide bonds formed between C134–C147 and C139–C145, whereas Ep2 is stabilized by a single disulfide bond (C172–C178). Ep1 forms a loop connecting helix α4 and β7 strand (Figure 2), whereas Ep2 forms a loop connecting β8 and β9 strands (Figure 2).

### 3.4. Epitope Peptide Synthesis

On the basis of in silico epitope predictions, we synthetized three N-terminally biotinylated epitope peptides for immune sera reactivity tests (ProteoGenix, Schiltigheim, France). 6-Aminohexanoic acid spacers were added between the biotin tag and the peptide (Smp1—TSYTSSSRDCKSRLNCQSNELLNSFMN; Smp2—TSYTCSSRDSKSRLNSQCNELLNSFMN, and Smp3—GKFVRTPGMCVLDRTCGTCE). Smp1 and Smp2 mimic epitope 1, while Smp3 is representative of epitope 2. To avoid mixed disulfide bond scrambling, given the presence of two disulfide bonds in Ep1, we synthesized two versions of the same peptide, containing the substitution of two different cysteine residues with serine residues (indicated in bold font and underlined). Synthetic peptides were slightly shortened to improve their solubility with respect to the full predicted sequences. Yet, the most important region *(*i.e., the reactive loops) was fully conserved. Unfortunately, due to the length of the peptide sequence, it was not practically feasible to synthesize a larger epitope encompassing both Ep1 and Ep2.

#### 3.4.1. Immune Sera Reactivity Tests Via DELFIA Assay

**Figure 3:**
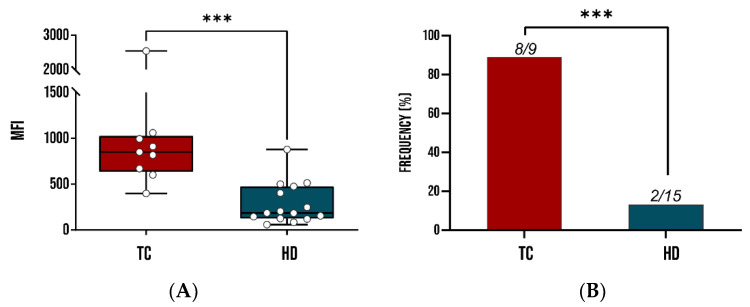
Immunoreactivity studies of recombinant *Tc*SMP in sera from patients infected by *T. cruzi* in comparison with healthy donors (HDs). A whisker plot comparing mean fluorescence intensity of mean fluorescence intensity (MFI) of *Tc*SMP tested against the sera of 9 *T. cruzi*-infected patients and 15 HDs. (**A**) Each dot represents the MFI of a single patient. (**B**) Recognition frequency of *Tc*SMP as determined by DELFIA assay. ***: statistical significance *p* < 0.0001 Student’s *t*-test and Fisher’s exact test, *p* < 0.0001 for MFIs and recognition frequencies. TC: *T. cruzi* patient sera; HD: healthy donors.

The association of recombinant *Tc*SMP with *T. cruzi* infection was assessed by DELFIA assay, as described in the Section 2. A comparative analysis showed that *Tc*SMP confirmed significantly higher IgG reactivity in patients than HD sera, showing a recognition frequency of 89% in terms of sensitivity and 87% in terms of specificity, as shown in Figure 3.

These results suggest that *Tc*SMP represents a potential serodiagnostic marker for *T. cruzi* infection.

#### 3.4.2. Polyclonal Antibody Recognition and Immune Sera Reactivity Tests Via Microarray

**Figure 4:**
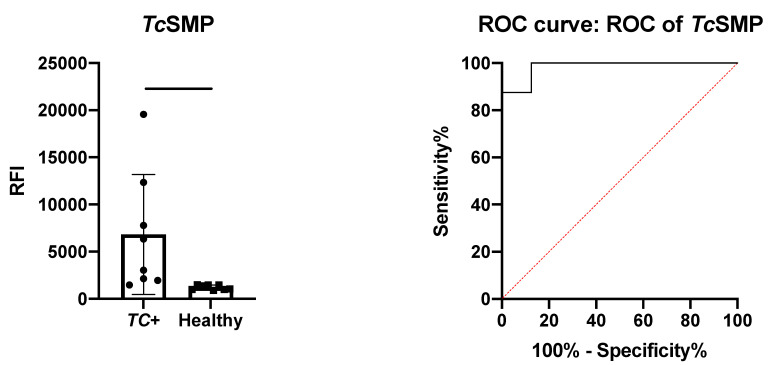
Immune sera reactivity potential of recombinant *Tc*SMP. Left panel: unpaired *t*-test results for the detection of *Tc*SMP-specific human IgG. Protein arrays were probed with sera (N = 8) from patients with confirmed *T. cruzi* infections (TC+) and healthy control patients (N = 14). Significative: *p* < 0.05.

The immunoreactivity of *Tc*SMP was assessed by protein microarray, as described in the Section 2. The protein microarray was incubated with the serum samples and with an anti-human IgG secondary antibody to check protein ability to detect serum antibodies from sera from both serum sample groups. As shown in Figure 4 (left panel), *Tc*SMP was recognized by human IgGs in sera from infected people (TC+), while the response of the protein to healthy serum was significantly lower (*p* = 0.0256). The area under the curve (AUC) after ROC analysis was 0.9844 (Figure 4, right panel). Even if it was based on a limited set of samples, the ROC curve analysis provided a *p*-value of 0.0011 (95% confidence interval) and, through setting a fluorescence threshold of 1478, 87.5% sensitivity and 100% specificity.

These data confirm that recombinant *Tc*SMP is a valid target for further development for serological-based immunodiagnostic tests for Chagas disease.

We previously demonstrated that peptide epitopes can possess improved immunological properties in comparison with the recombinant counterpart [22,56]. Furthermore, peptides can be specifically oriented to maximize antibody recognition, using eloquent chemical conjugation strategies [24,57,58,59]. In this context, we carried out structure-based in silico epitope predictions on the *Tc*SMP crystal structure, with the aim of designing epitope peptides as diagnostic biomarkers of CD.

**Figure 5:**
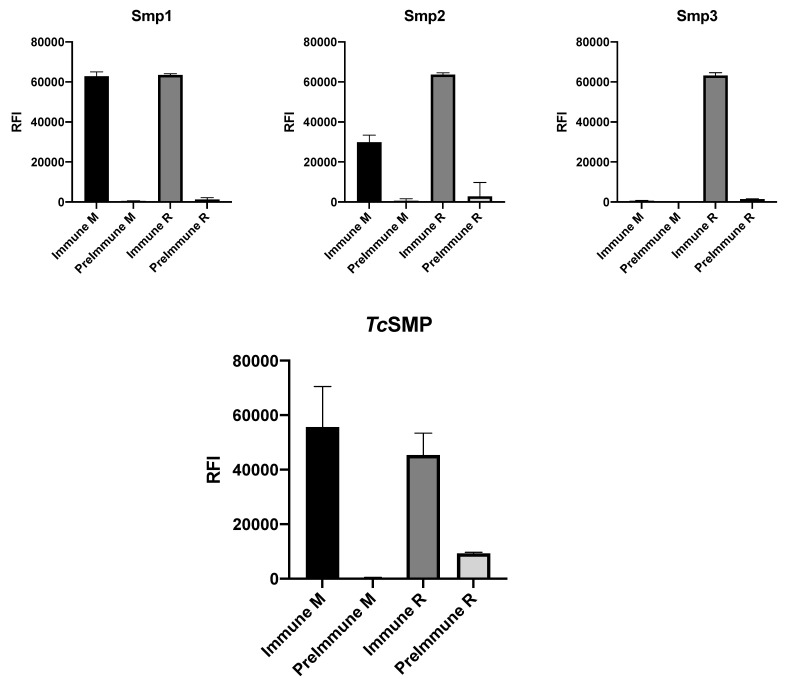
Immune sera reactivity of predicted epitopes. Upper panels: ability of biotinylated peptides Smp1, Smp2, and Smp3 to recognize antibodies present in mouse immune sera (M) and rabbit (R) immune sera, following immunization with recombinant *Tc*SMP. Lower panel: immune sera reactivity of mouse immune sera (M) and rabbit (R) immune sera, against the recombinant *Tc*SMP protein.

All three peptides house cysteine residues that may exist as reduced thiols or may participate in disulfide bond formation. To avoid disulfide bond scrambling, we mutated Smp1 and Smp2 to remove the C134–147 and C139–145 disulfide bonds, respectively, leaving only one of the two disulfide bonds present in the predicted epitopes. In contrast, three cysteine residues (C172, C178, and C181) were present in Smp3. Given such modifications, in order to confirm that epitope peptides adopt the same structural conformation reflected in the recombinant antigen, peptide microarrays displaying biotinylated peptides Smp1, Smp2, and Smp3 were probed with rabbit and mouse immune serum raised against *Tc*SMP (see the Section 2). Figure 5 compares peptide and recombinant protein immune sera reactivity of the immunized animals versus corresponding pre-immune sera controls. Fluorescence was detected after incubation with anti-rabbit and anti-mouse secondary antibodies labelled with Cy3.

Although immune sera from both rabbit and mouse recognized the recombinant antigen, and all three epitopes were recognized by immune rabbit serum, Smp3 was not immune-reactive against mouse serum (Figure 5). These data confirm that all three epitope peptides can adopt an ensemble of conformations that entails/mirrors that in the recombinant antigen; however, the differential recognition of Smp3 between mouse and rabbit sera underlies the immune variability that may be encountered between species. Moreover, the conformational variability accessible to the peptides may be instrumental in capturing subfamilies of polyclonal antibodies that may be elicited in response to transient conformations and that are present in the sera. In fact, a NCBI BLASTP 2.12.0 (https://blast.ncbi.nlm.nih.gov/Blast.cgi, accessed on 23 June 2021) of possible homologous mouse peptides revealed shared sequence identity to regions belonging to two mouse proteins. Residues 2–6 of Smp3 share 100% sequence identity with a zinc finger protein (sequence ID AAN52482.1), and residues 9–17 share 67% identity with NOD2 (sequence ID AAA37301.1); therefore, the explanation for the lack of reactivity against mouse sera is that the mouse immune system recognizes Smp3 as a self-epitope and thus does not produce antibodies against this epitope.

Although all three epitopes were recognized by rabbit and/or mouse immune sera raised against the recombinant protein, and recombinant *Tc*SMP was recognized by immune sera from human patients with confirmed *T. cruzi* infections, the three synthetic peptides were found not to be immunoreactive against human sera (data not shown) nor immunodominant epitopes in humans, or possibly Smp1 and Smp2 may constitute two parts of a larger conformational epitope, suggesting that further cycles of epitope re-design should be employed. In this context, the presence of cysteines could aptly be exploited to explore the impact of, e.g., conformational selection on human immunoreactivity by allowing simple and efficient cyclization (using suitable reaction conditions) or by providing points of attachment for further modification of the peptide mimics [22,58,59].

## 4. Conclusions

In short, we report the recombinant production of a protein antigen with confirmed application for the immune serum-based diagnosis of *T. cruzi* infections. We also provide a detailed analysis of the solved *Tc*SMP crystal structure, which represents only the second of its kind in the PDB. In the context of designing immunodiagnostic markers, we predicted the most antigenic (epitope) regions of the protein, using a structure-based computational prediction method. A particularly immunogenic hotspot was identified, harboring two epitopes that may compose a single, larger, conformational epitope. Designed epitope peptides were recognized by immune sera from mice and rabbits, confirming their ability to elicit antibodies and asserting their ability to adopt a 3D structure that is comparable to that in the whole protein. Although the immune sera reactivity of the whole recombinant protein has the future potential to be employed with other serodiagnostic antigens in a mutiplex diagnostic test, our data suggest that efforts should move in the direction of designing a more reactive recombinant domain, rather than synthetic epitope peptides, given the large conformational epitope that was detected in the in silico predictions.

## Data Availability

Not Applicable.

## Supplementary Materials

The following are available online at https://www.mdpi.com/article/10.3390/vaccines10010071/s1, Figure S1: (A) Akta FLPC chromatographic profile of His-trap (first step) purification and SDS-PAGE analysis of TcSMP (B) Size exclusion chromatography profile of TcSMP (second step) and SDS-PAGE analysis; Figure S2: Structural superposition of TcSMP with its two homologs; Figure S3: Root mean square fluctuation (RMSF) of *Tc*SMP (3 × 500 ns MD simulations); Table S1: Data collection and refinement statistics. Material and Methods: detailed multi-step minimization and equilibration procedure, and detailed MLCE method for epitope predictions.
