# Supplementary material for: Elucidating the 3D Structure of a Surface Membrane Antigen from Trypanosoma cruzi as a Serodiagnostic Biomarker of Chagas Disease
Source: Vaccines (Basel). 2022 Jan 3;10(1):71. doi: 10.3390/vaccines10010071 (PMC8781870; doi:10.3390/vaccines10010071)
Supplement: Supplementary file 1 [file vaccines-10-00071-s001.zip › vaccines-1508357-supplementary.pdf]

SUPPORTING INFORMATION

Elucidating the 3D structure of a surface membrane antigen from *Trypanosoma cruzi* as a serodiagnostic biomarker of Chagas disease.

Flavio Di Pisa [1] , Stefano De Benedetti [1§], Enrico Mario Alessandro Fassi [2,3], Mauro Bombaci [4], Renata Grifantini [4], Angelo Musicò [2], Roberto Frigerio [2], Angela Pontillo [5], Cinzia Rigo [6], Sandra Abelli [6], Romualdo Grande [7], Nadia Zanchetta [7], Davide Mileto [7], Alessandro Mancon [7], Alberto Rizzo[7], Alessandro Gori [2], Marina Cretich [2], Giorgio Colombo[2,8], Martino Bolognesi [1,9] Louise Jane Gourlay *[1].

***Tc*SMP Expression and purification – supporting figures**

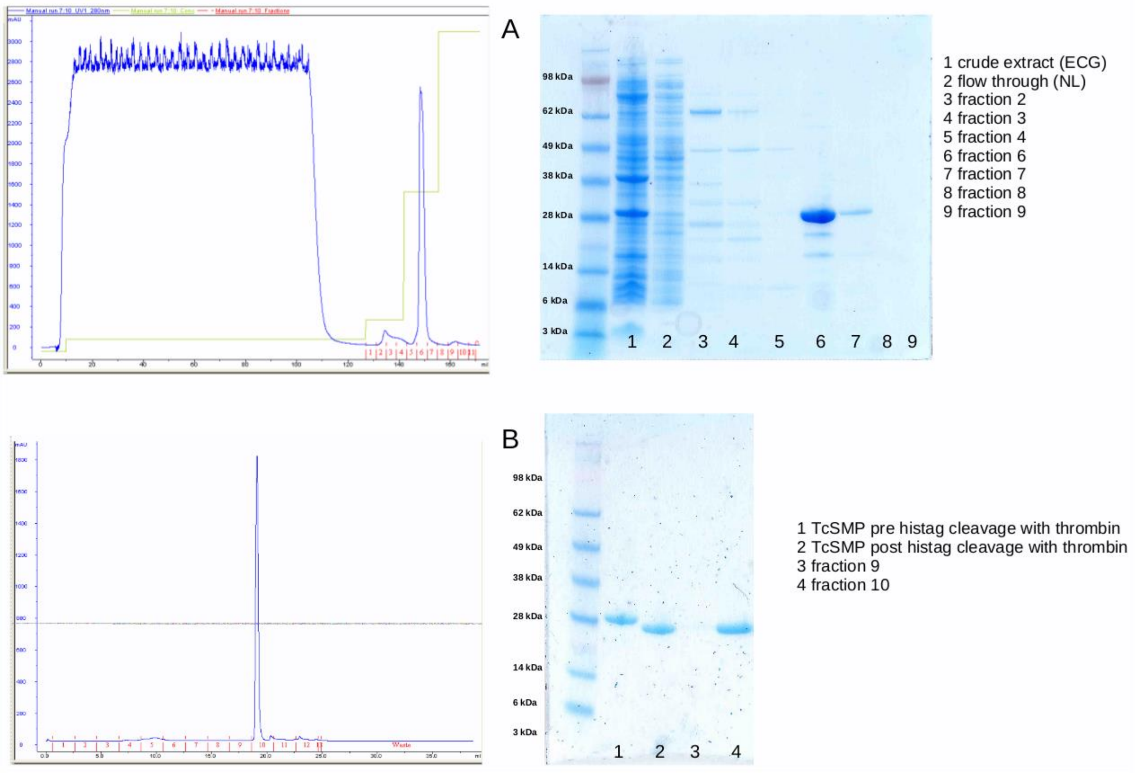

Figure S1. A) Akta FLPC chromatographic profile of His-trap (first step) purification and SDS-PAGE analysis of *Tc*SMP B) Size exclusion chromatography profile of *Tc*SMP (second step) and SDS-PAGE analysis.

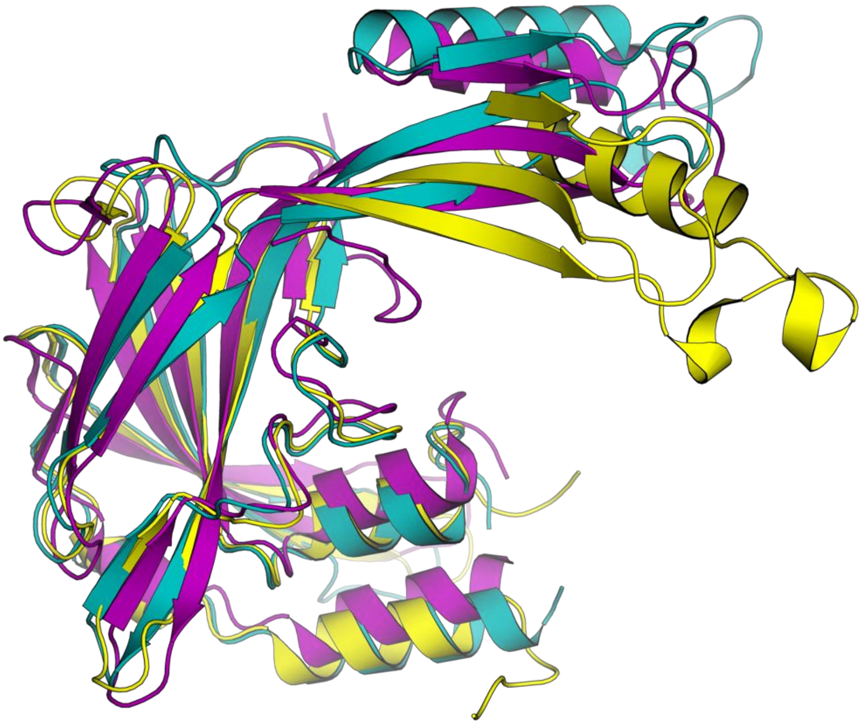

Figure S2. Structural superposition of *Tc*SMP with its two homologs: *T. brucei* procyclic-specific surface antigen-2 (pdb code 5KLH, yellow) and *T. congolense* insect stage antigen (pdb code 5KMX, purple) were superimposed on the *Tc*SMP crystal structure (teal). This image was generated using CCP4mg.

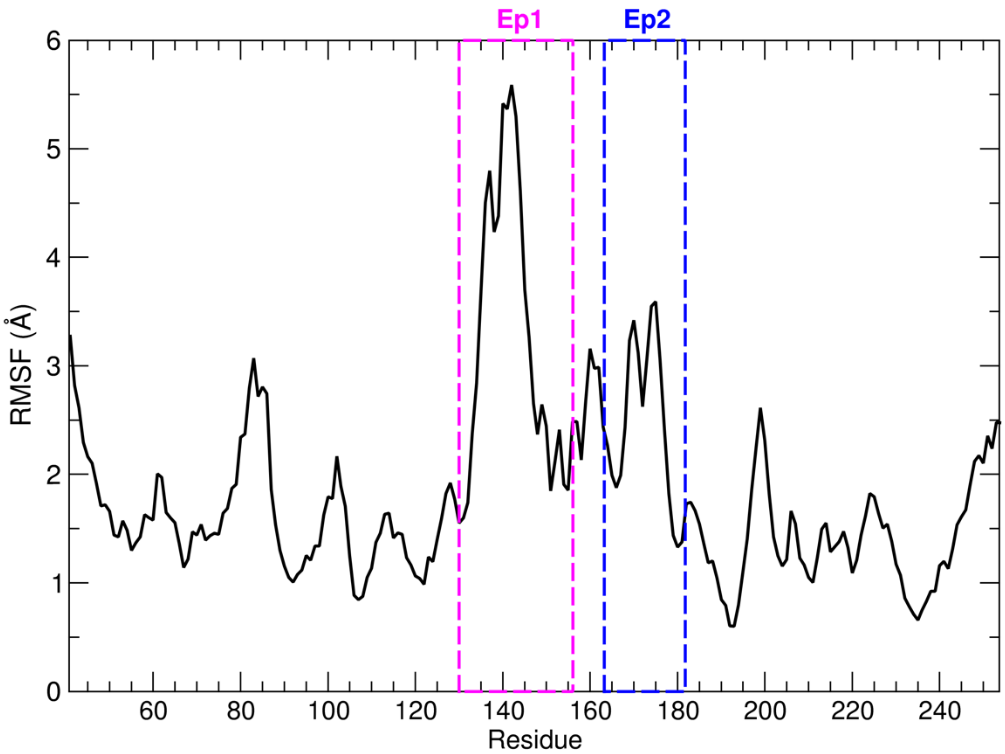

Figure S3. Root Mean Square Fluctuation (RMSF) of *Tc*SMP (3x500ns MD simulations). The predicted epitopes are showed in magenta and blue broken lines for Ep1 and Ep2, respectively.

X-ray data collection and refinement statistics

Table S1. X-ray diffraction data collected on a single crystal of TcSMP11.90. Values in parenthesis correspond to the high-resolution shell. For cross-validation, 5% experimental reflections were randomly selected to calculate the R_{free} value.

	TcSMP11.90<br>(PDB code 6Y0D)
**Crystal**	
Space group	C 1 2 1
Cell dimensions *a*, *b*, *c* (Å) ; ß (°)	95.89, 33.192, 67.445; 108.059
**Data collection**	
Beamline	DLS I04
Wavelength (Å)	0.9795
Resolution (Å)	64.12-1.62 (1.71-1.62)
Total reflections	161403 (17485)
Unique reflections	26050 (3749)
R_{merge}	0.059 (0.984)
#$R_{meas,}$	0.065 (1.111)
$I/\sigma(I)$	13.9 (1.5)
+$CC_{1/2}$	0.999 (0.694)

Completeness (%)	99.9 (99.6)
Redundancy	6.2 (4.7)
Wilson B-factor (Å)	25.38
**Refinement**	
Resolution (Å)	44.18-1.62
No. reflections	25643
R_{work} / R_{free}	19.2/22.9
No. atoms	
Protein	1641
Water	161
B factors	
Protein	38.63
Water	38.96
R.m.s. deviations	
Bond lengths (Å)	0.009
Bond angles (°)	0.95
Clash scores	2.81
Ramachandran	
Favored (%)	96.7
Allowed (%)	3.3

Redundancy-independent merging R factor R meas estimated by multiplying the conventional R merge value by the factor

[N/(N− 1)] 1/2 , where N is the data multiplicity.

+ CC 1/2 is the correlation coefficient of the mean intensities between two random half-sets of data.

Protein structure comparison

Dali server. Matches against all PDB structures (here reported the first 20, query submitted on 04/06/2021).

```
# Job: TcSMP 06042021
# Query: s001A
# No:  Chain   Z    rmsd lali nres  %id PDB  Description
   1:  5klh-B 25.7  4.1  196   215   40   MOLECULE: SURFACE GLYCOPROTEIN;
   2:  5kmx-A 24.8  2.7  194   202   41   MOLECULE: PUTATIVE UNCHARACTERIZED PROTEIN TCIL3000_10_9440
   3:  5klh-A 24.7  4.1  189   221   39   MOLECULE: SURFACE GLYCOPROTEIN;
   4:  5kmx-B 22.4  3.8  176   196   41   MOLECULE: PUTATIVE UNCHARACTERIZED PROTEIN TCIL3000_10_9440
   5:  5kmx-D 20.8  4.1  162   184   44   MOLECULE: PUTATIVE UNCHARACTERIZED PROTEIN TCIL3000_10_9440
   6:  5kmx-C 19.6  4.3  178   182   42   MOLECULE: PUTATIVE UNCHARACTERIZED PROTEIN TCIL3000_10_9440
   7:  5vli-C  4.4  1.6   37    39    5   MOLECULE: HEMAGGLUTININ;
   8:  3hoi-A  4.2  4.3   61   193    2   MOLECULE: FMN-DEPENDENT NITROREDUCTASE BF3017;
   9:  4yut-A  4.0  3.6   61   351   11   MOLECULE: FAMILY 3 ADENYLATE CYCLASE;
  10:  3ucq-A  3.9  6.8   83   651   10   MOLECULE: AMYLOSUCRASE;
  11:  2gjh-A  3.5  4.7   53    57    9   MOLECULE: DESIGNED PROTEIN;
  12:  5jg9-B  3.5  2.4   43    47    5   MOLECULE: DE NOVO DESIGN, HYPER STABLE, DISULFIDE-RICH MINI
  13:  4fls-A  3.5  5.9   87   628   10   MOLECULE: AMYLOSUCRASE;
  14:  1mw3-A  3.5  5.7   85   628    9   MOLECULE: AMYLOSUCRASE;
  15:  7jh4-A  3.4 11.6   55   223   11   MOLECULE: NAD(P)H-DEPENDENT OXIDOREDUCTASE;
  16:  4kyz-A  3.4  7.0   72   167   11   MOLECULE: DESIGNED PROTEIN OR327;
  17:  1zs2-A  3.4  6.3   81   628   11   MOLECULE: AMYLOSUCRASE;
  18:  5mbh-A  3.4 18.0   72   339   11   MOLECULE: BETA SUBUNIT OF PHOTOACTIVATED ADENYLYL CYCLASE;
  19:  5xwk-A  3.2  7.2   89   530    9   MOLECULE: ALKALINE PHOSPHATASE PHOK;
  20:  7c50-A  3.2  7.6   79   192    4   MOLECULE: SIMPL DOMAIN-CONTAINING PROTEIN;
```

Material and Methods: Detailed multi-step minimization and equilibration procedure

The solvated system was relaxed by a two-step protocol to remove atomic clashes: firstly, we performed an energy minimization for 10,000 steps, or until the energy gradient of 0.002 kcal/mol/Å was reached, applying a harmonic potential restraint to the backbone atomic coordinates (k = 20 kcal/mol/Å^2). Next, we performed an energy minimization

for 20,000 steps, or until an energy gradient of 0.002 kcal/mol/Å was reached, without applying any restraint. After the minimization procedure, the system was equilibrated gradually increasing the temperature to 200 K over 200 ps under constant volume condition (NVT). Finally, the system was progressively heated to 300 K and equilibrated for 100 ps under constant pressure conditions (NPT, 1 atm).

Material and Methods: Detailed Prediction of epitopes: MLCE

MLCE is a technique based on the analysis of the interaction energies of all the amino acids in a protein. In particular, it computes the non-bonded part of the potential (van der Waals, electrostatic interactions, solvent effects) via a MM/GBSA calculation, obtaining, for a protein composed by N residues, a N×N symmetric interaction matrix M_{ij}. This matrix can be expressed in terms of its eigenvalues and eigenvectors as

$$M_{ij} = \sum_{\alpha=1}^{N} \lambda_\alpha \, v_i^\alpha v_j^\alpha$$

where λ_α is the α-th eigenvalue and v_i^α is the i-th component of the corresponding eigenvector. The eigenvector with the most negative correspondent eigenvalue contains most of the interaction information for the stabilizing interaction of the system. An approximated interaction matrix $M\sim_{ij}$ is thus given by

$$M\sim_{ij} = \lambda_1 v_i^1 v_j^1$$

If the structure of the protein is known, one can estimate a contact matrix C_{ij} by assuming two amino acids in contact if the distance between two of their heavy atoms is smaller than a threshold. The Hadamard product of the two matrices gives us the matrix of the local coupling energies.

$$MCLE_{ij} = M\sim_{ij} \cdot C_{ij}$$

We select as possible interacting zones sets of close by residues that show weak or frustrated interactions.

The analysis of the energetic properties of the surface residues is based on the MLCE method. Basically, we perform a MM/GBSA analysis of the structure in a force field, obtaining a symmetric per-residue interaction matrix M_{ij} keeping only non-bonded interaction (i.e. electrostatic, van der Waals and solvation contributions). We diagonalize the matrix, obtaining a set of eigenvectors $x^{(i)}$ sorted following the increasing value of their eigenvectors λ_i where N is the number of amino acids in the sequence. We thus can write the original matrix M_{ij} as

$$M_{ij} = \sum_{k=1}^{N} \lambda_k \, x^{(k)}(i) x^{(k)}(j)$$

It has been shown that the first eigenvector alone can be used to build an approximate interaction matrix $M : M_{ij} = \lambda_1 x^{(1)}(i) x^{(1)}(j)$, which recapitulates the interactions most relevant for the stabilization of a certain conformation of a defined protein or protein substructure.

The MM/GBSA is performed with Amber 14 software using the ff14SB forcefield.
